# Adolescence in the digital age the influences of smartphone and social media use on a cohort of Irish teenagers

**DOI:** 10.1192/j.eurpsy.2025.355

**Published:** 2025-08-26

**Authors:** F. Donnelly

**Affiliations:** 1 National Health Service Improvement, Health Service Executive, Dublin, Ireland

## Abstract

**Introduction:**

The aim of this study was to investigate the associations between smartphone and social media use and mental health outcomes among adolescents in Ireland.

**Objectives:**

This study aimed to fill a gap in the existing literature by examining specific types of online activity, such as cyberbullying and “sexting” (sending sexually explicit messages), and the independent associations of these activities with worse mental health.

**Methods:**

This study is a cross-sectional analysis of secondary data from the 2023 Planet Youth Partner survey. The study population was 4,544 mostly 15- and 16-year olds from Cavan, Monaghan and North County Dublin. The dependent variable was the total Strengths and Difficulties Questionnaire (SDQ) score, a tool commonly used to assess mental health status. Independent variables included hours of social media use, experiences of cyberbullying, body image perceptions, and involvement in sexting. Multivariable logistic regression models were employed to determine the associations between these variables and mental health outcomes, controlling for potential confounders such as gender, sleep duration, and maternal education level.

**Results:**

The study found significant associations between several online activities and worse mental health. High use of social media (4 hours per day or more) was associated with a 62% greater risk compared to those who used it for about 1 hour or less.

Cyberbullying was a also strong predictor of poor mental health, with victims nearly twice as likely to have a high SDQ score. Negative perceptions of one’s own body image and participating in sexting were also significantly associated with worse mental health outcomes.

Gender differences were observed, with females more likely to be victims of cyberbullying, to be asked for and to send sexually explicit messages, and to have worse mental health outcomes when compared to males. Insufficient sleep was also a significant predictor, with those sleeping for 6 hours or less per night having more than twice the risk compared to those who slept for the recommended 8 hours or more.

**Conclusions:**

The findings of this study suggest that specific aspects of smartphone and social media use, particularly high usage and negative online experiences, are independently associated with poorer mental health outcomes in adolescents. These results are consistent with international evidence and highlight the need for targeted public health interventions to mitigate the risks associated with this technology. The study recommends developing evidence-based guidelines for parents and teachers to promote healthier online behaviours among adolescents in Ireland. It also calls for a national Public Health campaign and policy measures to enforce stricter regulations on social media companies and protect young people from harmful online experiences.

**Disclosure of Interest:**

None Declared

